# Hydrothermal synthesis of nanohydroxyapatite-activated carbon composites and its slow-release performance for urea

**DOI:** 10.1038/s41598-025-09023-w

**Published:** 2025-07-19

**Authors:** Sabila Aulia Hemzah, Irwan Kurnia, Diana Rakhmawaty Eddy, Bedah Rupaedah, Suryana, Azman Bin Ma’Amor, Guoqing Guan, Atiek Rostika Noviyanti

**Affiliations:** 1 Department of Chemistry, Faculty of Mathematics and Natural Sciences, Universitas Padjadjaran, Sumedang, 45363 Indonesia; 2 Research Center for Applied Microbiology, National Research and Innovation Agency, Bogor, 16911 Indonesia; 3 Department of Biology, Faculty of Mathematics and Natural Sciences, Universitas Padjadjaran, Sumedang, 45363 Indonesia; 4 Department of Chemistry, Universiti Malaya, Kuala Lumpur, 50603 Malaysia; 5 Institute of Regional Innovation, Hirosaki University, Hirosaki, 036-8561 Japan; 6 Research Collaboration Center for Microbial Nanomaterials between BRIN and Universitas Padjadjaran, Sumedang, 45363 Indonesia

**Keywords:** Nanohydroxyapatite, Activated carbon, Composite, Urea, Slow release material, Sustainability, Other nanotechnology

## Abstract

Urea is the most widely used nitrogen-based fertilizer, but its excessive application leads to nutrient loss through leaching and evaporation. This study presents a nanohydroxyapatite (nHA)–activated carbon (AC) composite synthesized via hydrothermal method as a slow-release material (SRM) for urea. The composites of nHA–AC (HC) enhances urea adsorption and release control. Structural and functional group of composites were confirmed by XRD and FTIR, while TEM revealed nHA nanorods coating the surface of AC. Furthermore, zeta potential and surface area of HC showed a better properties than pure nHA. The composite HC1 (nHA:AC = 75:25) showed the best performance, releasing only 17% of urea over 240 min and adsorbing 0.54 g_urea_/g_SRM_ (36.2%). These results highlight the potential of HC1 as an efficient slow-release fertilizer to improve nutrient use efficiency in agriculture.

**Supplementary Information:**

The online version contains supplementary material available at 10.1038/s41598-025-09023-w.

## Introduction

In agricultural systems, excessive fertilizer application is shared to improve soil quality and crop yields. However, overuse of fertilizers reduces the efficiency of nutrient absorption by plants, commonly referred to as Nutrient Use Efficiency (NUE). NUE represents the relationship between the amount of nutrients plants absorb and the resulting biomass output^1,2^. Urea, a widely used nitrogen fertilizer, has an NUE of only 50% because 2–20% of the fertilizer is lost through volatilization, 15–25% reacts with organic compounds in the soil, and 2–10% is lost through leaching due to heavy rainfall or irrigation^3–5^. The slow diffusion of nutrients and their strong affinity for binding to inorganic and organic soil particles further limit plant nutrient uptake, leading to micronutrient deficiencies. As a result, fertilizers are often applied in excess^6^. This excessive application contributes to nutrient leaching and accumulating unabsorbed fertilizers, which are significant causes of eutrophication and environmental pollution^7–10^. Strategies such as slow-release fertilizers are needed to mitigate these issues to enhance fertilizer use efficiency.

Slow-release fertilizer is an advanced technology designed to provide nutrients to plants gradually. This technology releases nutrients to plant needs while minimizing leaching, thereby improving fertilizer use efficiency, reducing nutrient losses, extending nutrient availability, decreasing application frequency, and minimizing environmental pollution^11–14^. Slow release is achieved by coating fertilizers with hydrophobic materials or forming composites incorporating urea into molecular structures, thereby controlling nutrient release^15–18^. One of the materials that has the potential for slow release of urea is hydroxyapatite (Ca_10_(PO_4_)_6_(OH)_2_).

Many researchers have formulated slow-release fertilizers by combining hydroxyapatite (HA) and urea to improve plant nutrient delivery. Hydroxyapatite (HA) is a potential material for this application as it is biocompatible, biodegradable, and can store and release nutrients gradually^19–22^. Nanohydroxyapatite (nHA) are a continuous source of calcium and phosphate micronutrients and can be used in surface modification for nanohybrid formation^23^. Previous research explored nHA surface modification with urea and *Gliricidia sepium*, resulting in a nano-fertilizer that demonstrated slower nitrogen release than single urea fertilizer^10^. Another study developed nHA as a novel phosphorus fertilizer, reporting a 32.6% increase in plant growth rate and a 20.4% increase in soybean yield compared to conventional fertilizers. Furthermore, nHA-based fertilizers are environmentally friendly and do not exhibit toxic effects on seed germination^24^.

To enhance the specific properties of nanohydroxyapatite (nHA), it can be composited with other materials such as chitosan, activated carbon, or polymers to form nHA composites^25–30^. Several studies have reported on nHA composites with various materials. Channab et al.,2023 developed a carboxylated hydroxyapatite-cellulose composite as a slow-release nitrogen fertilizer by immobilizing urea within the composite. Functionalization of cellulose with carboxyl groups enhanced the interaction between the composite and urea, improving nitrogen retention and prolonging release kinetics^31^. Long et al., 2019 also synthesized nHA-coated activated carbon for Pb(II) removal from aqueous solutions. Their results showed that the nHA-activated carbon composite exhibited a maximum Pb(II) adsorption capacity of 416.67 mg/g, significantly higher than many other adsorbents. Among various materials composited with nHA, activated carbon is versatility due to its adaptability, high adsorption capacity, and non-toxicity^32^.

Activated carbon is an adsorbent that is often used because it has a well-developed pore structure, large surface area, and high level of surface reactivity^33–36^. Morphologically, its porous structure is highly effective in binding and storing soil nutrients, releasing them gradually according to plant consumption rates. A study on activated carbon derived from corn cobs demonstrated its potential as a carrier material for slow release microfertilizers, specifically for Cu²⁺, Fe²⁺, and Zn²⁺. The results highlighted its favourable characteristics, including high adsorption capacity and surface area^37^.

Nanohydroxyapatite (nHA) combined with carbon materials has potential applications across various industrial sectors. One of the widely used applications is the adsorption of organic molecules. Fernando et al., 2015 investigated Pb²⁺ adsorption using activated carbon and HA composites^38^. Similarly, Ferri et al., 2021 reported that HA-AC composites effectively adsorb both organic (methylene blue) and inorganic (Cu²⁺ and Ni²⁺) pollutants^39^. However, the application of nHA-activated carbon composites as a macro-nutrient gradual-release material for urea has not been explored, particularly in enhancing adsorption capacity and improving urea slow-release efficiency.

Based on this background, this study aims to develop a nanohydroxyapatite-activated carbon composite as slow-release materials (SRMs) for urea fertilizer with high adsorption capacity. The composite was synthesized using a hydrothermal method, with nHA sourced from chicken eggshells. This method was selected for its advantages: low cost, rapid reaction steps, low-temperature synthesis, ease of use, and high-purity nHA production^40^. The synthesized composite was characterized for its elemental composition and structure using X-ray fluorescence (XRF), X-ray diffraction (XRD), and Fourier transform infrared (FTIR) spectroscopy. Surface charge was analyzed using zeta potential measurements, while morphology and surface characteristics were examined using a transmission electron microscope (TEM) and a surface area analyzer (SAA), respectively. A UV-visible spectrophotometer was also used to assess urea’s adsorption and release capacity.

## Results and discussion

### Materials characterizations

**Fig. 1:**
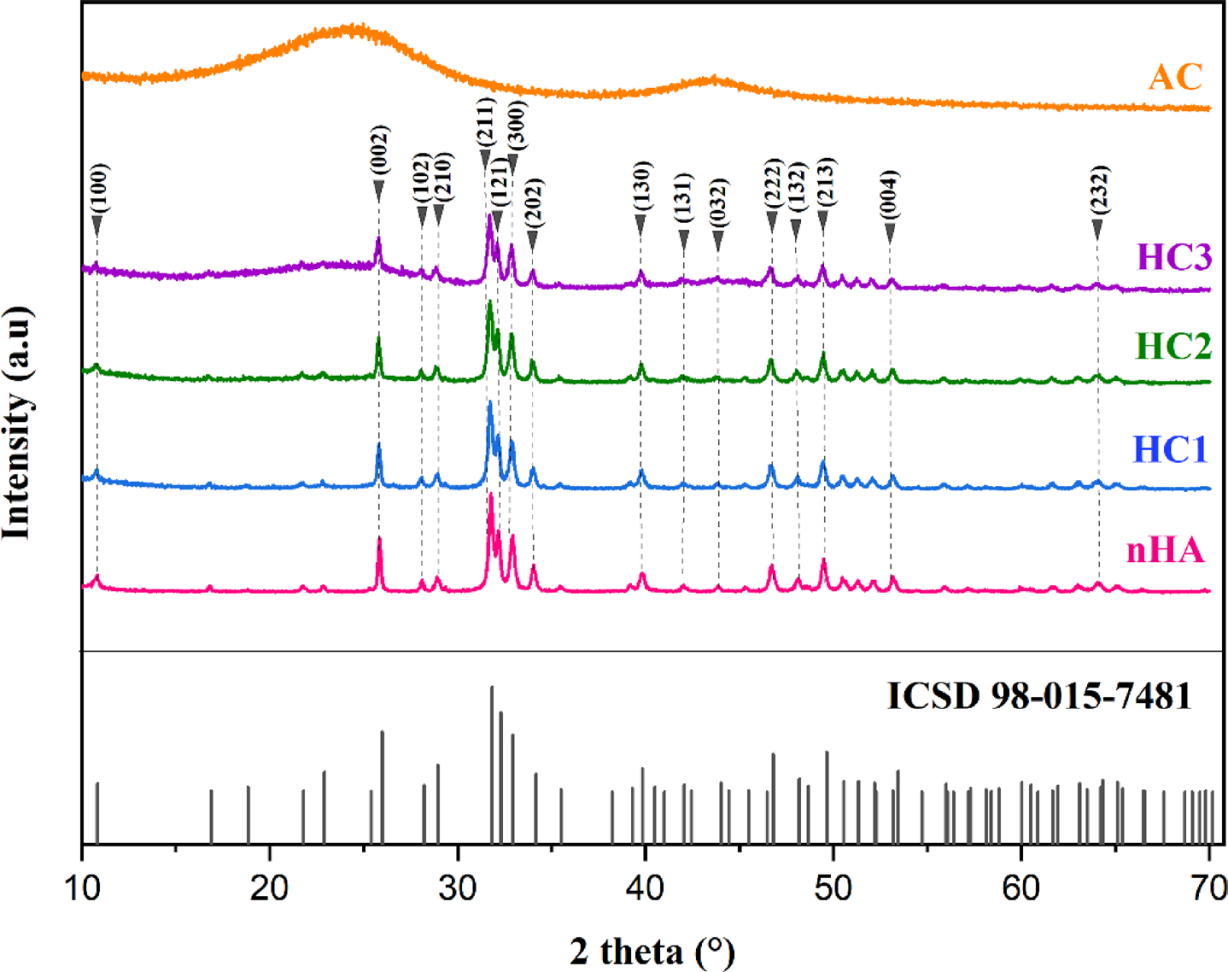
XRD structural patterns of hydroxyapatite (ICSD 98-015-7481 standard), nanohydroxyapatite (nHA), nanohydroxyapatite-activated carbon composites (HC1, HC2, and HC3), and activated carbon (AC).

Figure 1 illustrates the XRD patterns of all samples in the 2θ range of 10° to 70°. The XRD analysis confirms nanohydroxyapatite (nHA) was successfully synthesized with a nanoscale hexagonal crystal structure (space group P6₃/m), consistent with the ICSD 98-015-7481 standard. The characteristic diffraction peaks of nHA appear at 2θ = 10.79°, 25.82°, 28.07°, 28.15°, 28.90°, 34.02°, 39.78°, 41.97°, 42.29°, 46.67°, 48.06°, 49.44°, and 55.85°, in agreement with previously reported nHA crystal structures^41,42^. Meanwhile, activated carbon (AC) exhibits two broad diffraction peaks at approximately 2θ = 24° and 44°^43,44^, indicating its amorphous nature. For all three composites (HC1, HC2, and HC3), a combination of crystalline nHA and amorphous AC characteristics is observed through variations in diffraction peak intensity. A higher nHA ratio results in sharper diffraction peaks, indicating increased crystallinity, while an increase in AC content enhances the presence of amorphous features.

**Fig. 2:**
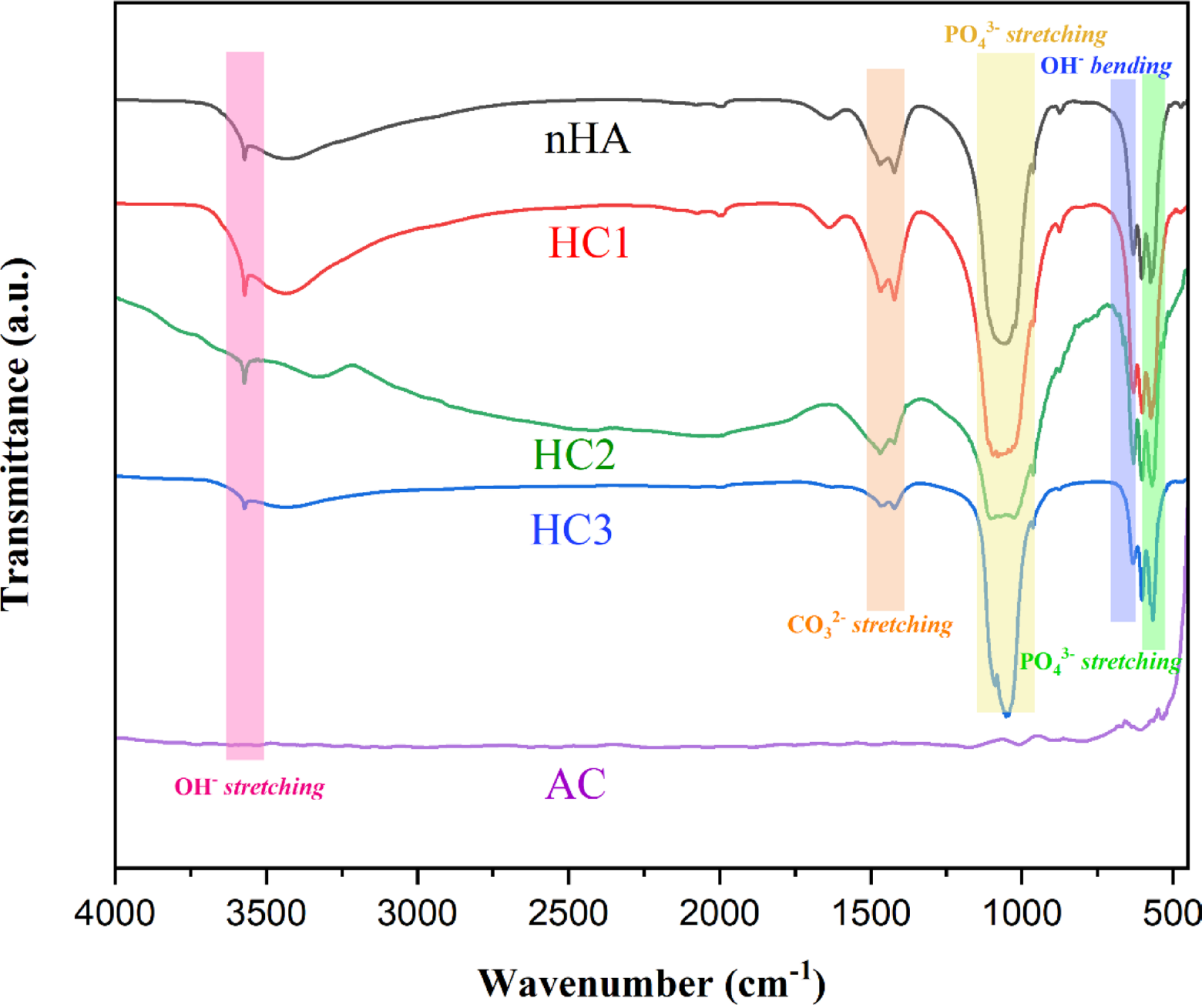
FTIR spectra of nanohydroxyapatite (nHA), nanohydroxyapatite-activated carbon composites (HC1, HC2, and HC3), and activated carbon (AC).

FTIR analysis confirmed the presence of characteristic functional groups of nHA and AC within the composites, as shown in Fig. 2. The nHA spectrum displayed absorption bands at 3571 cm⁻¹ (O–H stretching), 631 cm⁻¹ (O–H bending), 1056 cm⁻¹ and 963 cm⁻¹ (P–O stretching of phosphate groups), and 603 cm⁻¹ (P–O bending of phosphate groups). The FTIR spectra of the composites (HC1, HC2, and HC3), revealed absorption bands corresponding to the functional groups of both nHA and AC, confirming their successful incorporation. Although no new chemical bonds were detected between nHA and AC, both components’ presence in the composite were validated. These findings were further corroborated by XRD and TEM analyses, confirming the successful synthesis of nHA-AC composites.

**Fig. 3:**
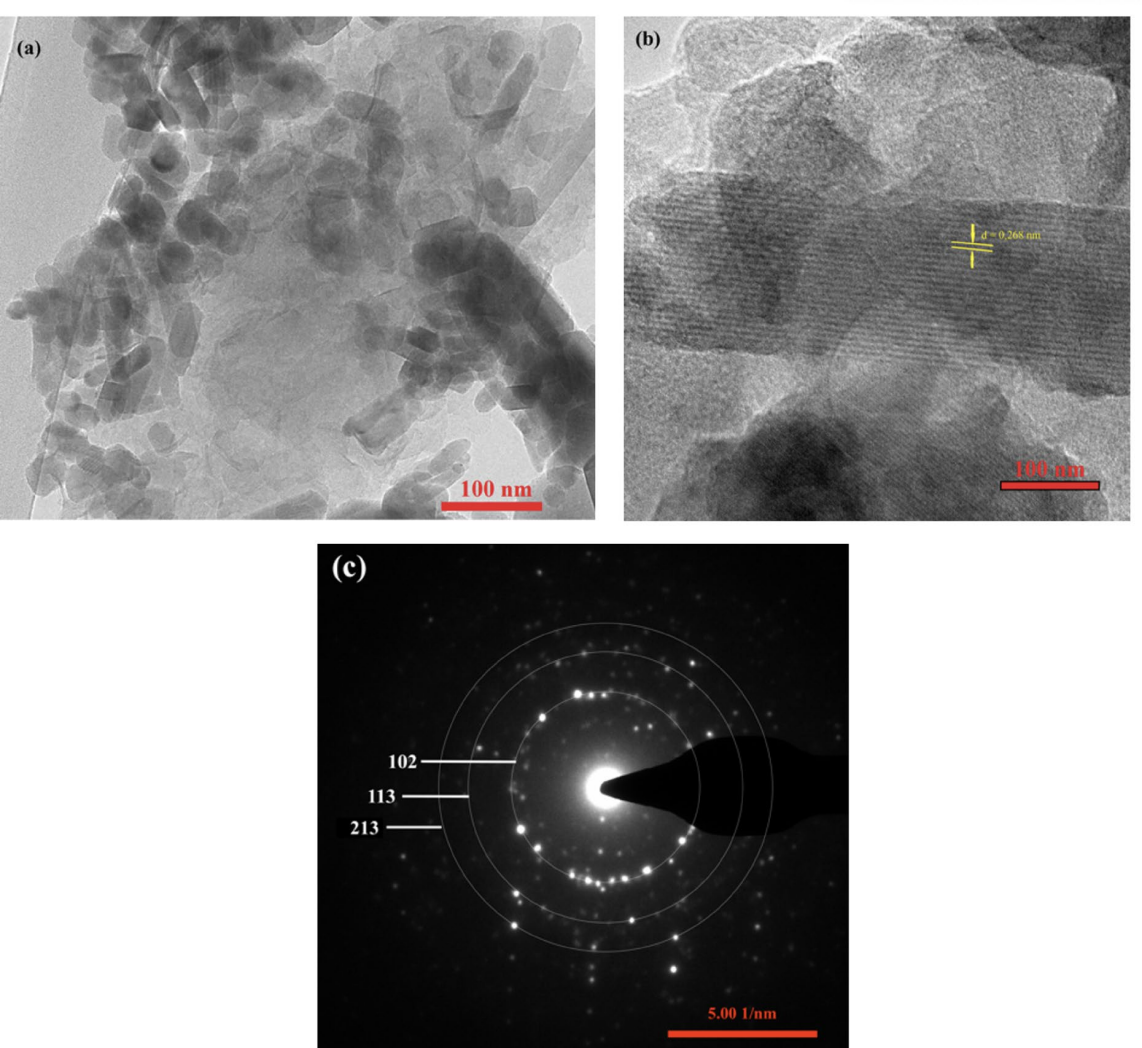
Morphology of HC1 composite (**a**), HR-TEM image of HC1 composite (**b**), and SAED pattern of HC1 composite (**c**).

Figure 3 presents TEM analysis results, demonstrating that nHA has a nanorod morphology with diameters ranging from 15 to 25 nm and lengths between 100 and 200 nm. The crystal structure of HA nanorods coated with activated carbon exhibits a lattice spacing of 0.268 nm, corresponding to the (121) plane of hexagonal nHA. Selected area electron diffraction (SAED) pattern confirms the polycrystalline nature of nHA, with diffraction peaks corresponding to the (102), (113), and (231) planes.

### Relation of surface properties and adsorption capacity of SRMs

Specific surface area (SSA) and zeta potential are surface properties that affect the urea adsorption capacity of a material^45–47^. The adsorption test results are presented as mean ± standard deviation and can be seen in Figure S3 and Table S4. Table 1 shows each SRM’s surface area, zeta potential, and adsorption capacity. The results show that the HC1 composite has the highest adsorption capacity (0.5431 g_urea_/g_SRM_) compared to other composites, but it is still lower than pure nHA and higher than AC. The HC1 composite had the lowest SSA and highest zeta potential (i.e., least negative) compared to the other composites. In this experiment, urea solution was used to evaluate the adsorption capacity of nanohydroxyapatite (nHA), as a model of SRMs (Table S2, entries 2–4). The effectiveness of nHA in adsorbing urea is attributed to the presence of Ca²⁺ ions that can interact with urea through electrostatic bonding, as supported by the zeta potential analysis showing that nHA has a more favorable surface charge than AC and the composites. Although AC has the largest specific surface area, its urea adsorption capacity is lower because it does not have active sites that can interact specifically with urea. These results suggest urea adsorption is more influenced by zeta potential than SSA, where materials with more positive zeta potential, such as nHA, can attract urea molecules more strongly than AC, which has more negative zeta potential. The specific active sites in nHA make nHA adsorb more urea^38^. Therefore, zeta potential is more dominant in determining urea adsorption efficiency based on composite materials than on a specific surface area.

Specific surface area (SSA) and zeta potential are key surface properties influencing the urea adsorption capacity of a material^45–47^. Table 1 presents each sample’s SSA, zeta potential, and adsorption capacity. The results indicate that the HC1 composites exhibit the highest adsorption capacity (0.5431 g_urea_/g_SRM_). However, its adsorption capacity remains lower than that of pure nHA but higher than that of AC. Interestingly, the HC1 composite also possesses the lowest SSA and the highest (least damaging) zeta potential among all samples. nHA’s high urea adsorption efficiency is attributed to Ca²⁺ ions, which facilitate electrostatic interactions with urea. This finding is further supported by zeta potential analysis, revealing that nHA has a more favorable surface charge than both AC and the composites. While AC has the highest SSA, its lower urea adsorption capacity is likely due to the absence of specific active sites capable of interacting with urea molecules. These results suggest that zeta potential is more dominant than SSA in urea adsorption. Materials with a more positive zeta potential, such as nHA, exhibit stronger electrostatic attraction toward urea than AC, which has a more negative zeta potential. Specific active sites in nHA further enhance its urea adsorption capacity^48^. Therefore, zeta potential is a critical factor in determining the adsorption efficiency of composite materials for urea.

**Table 1:** Adsorption capacity, specific surface area (SSA), and zeta potential of nHA, AC, and composites (HC1, HC2, and HC3).

Sample	% Urea adsorbed	Specific Surface Area (m^2^/g)	Zeta potential (mV)
nHA	39.08	45.35	−33.5
HC1	36.20	124.63	−34.5
HC2	30.66	235.60	−36.1
HC3	27.34	425.40	−42.2
AC	24.24	514.31	−55.8

The FTIR spectrum provides clear evidence of urea loading on each sample, as shown in Figure S2 and Table S1. Characteristic urea absorption bands at 1720–1660 cm⁻¹ (C = O stretching) and 3475–3400 cm⁻¹ (N-H stretching) are observed in nHA, AC, and the composites following the adsorption process. The C = O stretching band exhibited an increased intensity and shift for nHA, indicating a strong interaction between urea and Ca²⁺ ions on the nHA surface. In contrast, AC displayed a weaker C = O absorption band, suggesting lower adsorption efficiency due to the absence of specific interactions with urea. In the nHA-AC composites, the intensity of the C = O band varied depending on the nHA-to-AC ratio, with composites containing a higher proportion of nHA demonstrating more significant urea adsorption. These results confirm urea adsorption is governed by surface properties and chemical interactions, with nHA playing a dominant role in the adsorption mechanism.

### Evaluation of slow-release properties for Urea

**Fig. 4:**
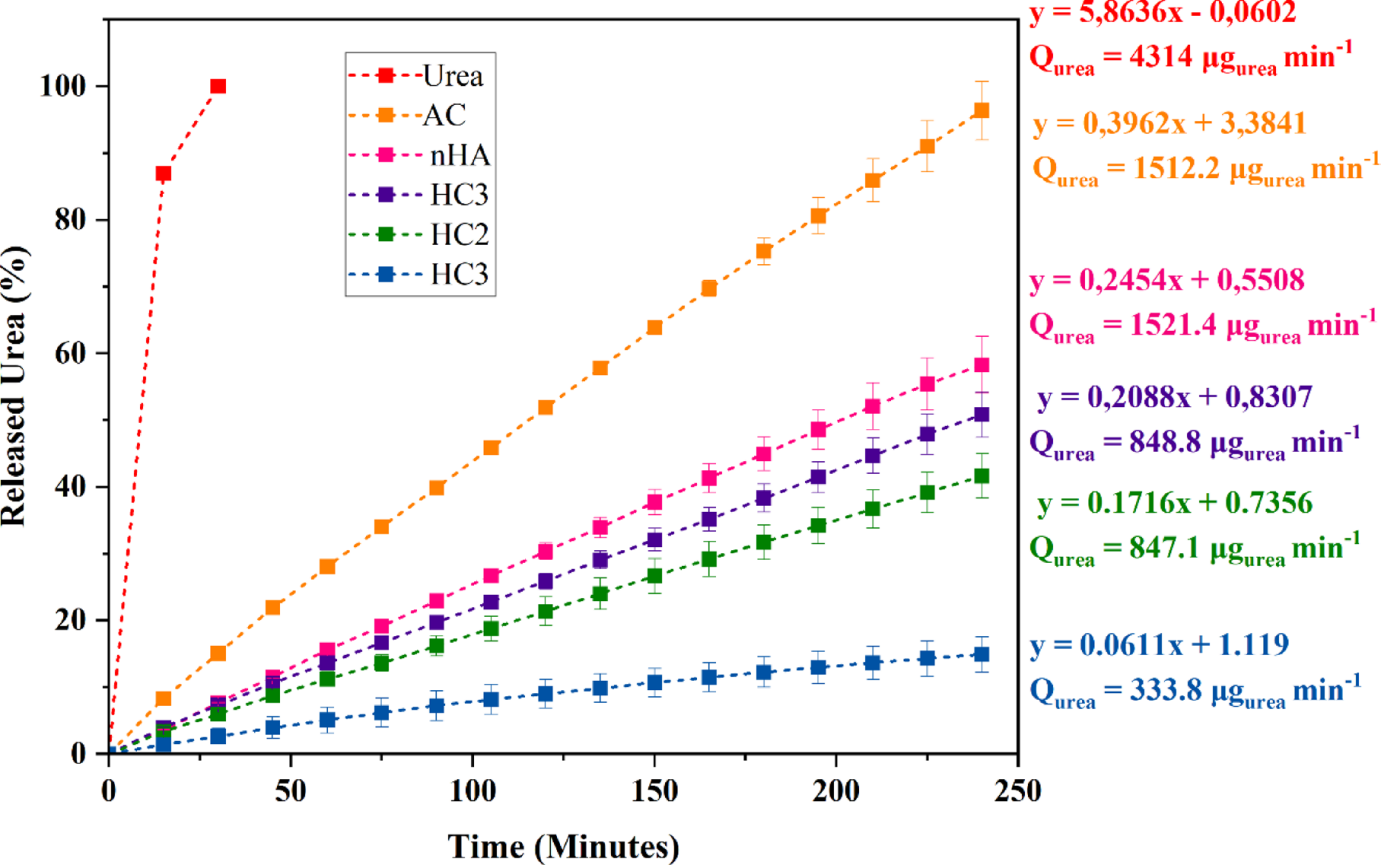
Urea release test on nanohydroxyapatite (nHA), nanohydroxyapatite-activated carbon composites (HC1, HC2, and HC3), and activated carbon (AC).

The urea release test was conducted to evaluate the effectiveness of each material in controlling the gradual release of urea. This test simulated environmental conditions using a column filled with silica sand as a simplified soil matrix, with distilled water supplied at a flow rate of 5 mL/min for 240 min (4 h) to mimic the dissolution of urea by rainwater. However, The release rate equations, urea release rates, and the estimated times required for complete urea release for each sample are summarized in Table S3. The cumulative urea release percentage over time is illustrated in Fig. 4 and summarized in Table S5, with the results reported as mean ± standard deviation.

**Fig. 5:**
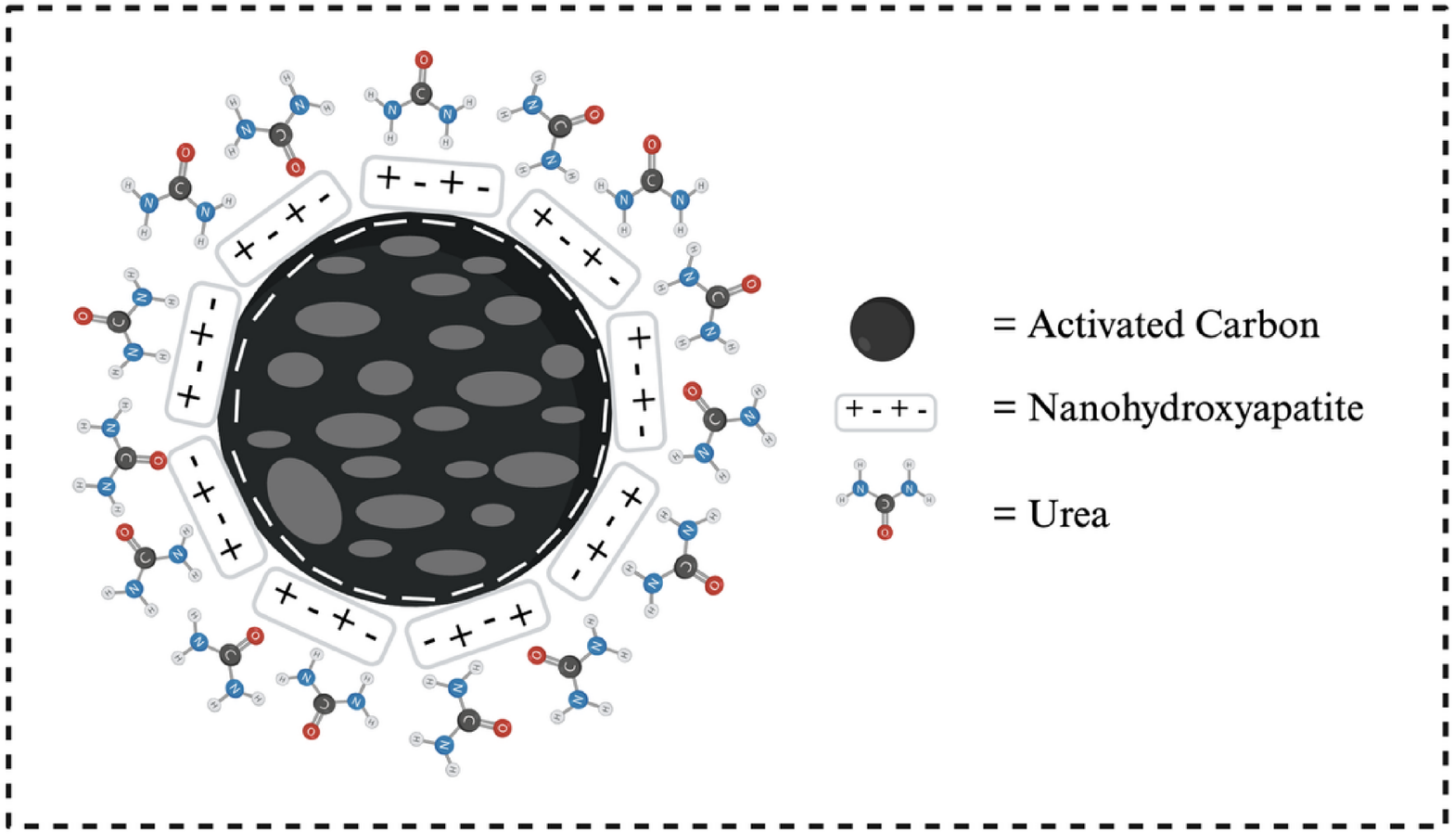
Synergistic interaction between nHA-AC-urea in HC1.

As shown in Fig. 4, pure urea dissolved rapidly, with almost 100% release within 30 min, indicating its high solubility and susceptibility to leaching. Activated carbon (AC) adsorbed urea releases 96% of its adsorbed urea within 240 min, indicating its limited effectiveness in inhibiting urea release. In contrast, nanohydroxyapatite (nHA) exhibited a slower release rate, with 58% of urea released over the same period. This behavior can be attributed to the interaction between Ca²⁺ and PO₄³⁻ ions on the nHA surface and urea molecules, forming temporary bonds that slow the release process. Among the three nanohydroxyapatite-activated carbon composites (HC1, HC2, and HC3), urea release was significantly slower than in nHA and AC alone, with the release percentages ranked as follows: HC1 (15%), HC2 (42%), and HC3 (51%). The trend suggests that a higher nHA content in the composite enhances urea retention due to more active sites capable of electrostatic interactions with urea molecules. The synergistic interaction between nHA-AC, as depicted in Fig. 5. As previously mentioned, the zeta potential of the SRM significantly influences its urea sorption capacity. Although nHA exhibits a higher zeta potential, which facilitates urea binding, its nanoscale size makes it prone to leaching by water. In contrast, the HC1 composite contains AC and has a lower zeta potential than pure nHA. This allows the AC component to function as an anchoring matrix, reducing the mobility of the nHA–urea complex and minimizing its dissolution (Fig. 5).

All three composites combine physical and chemical-based fertilizers, where AC serves as a structural buffer to slow release. At the same time, nHA plays a primary role in adsorption. The HC1 composite emerged as the most effective candidate, demonstrating the slowest urea release and the highest adsorption capacity. This suggests its potential as an optimized material for slow-release fertilizers. Compared to nHA and AC individually, the significantly reduced urea release rate from the nHA-AC nanocomposite indicates that the synergistic combination of these materials enhances release control. This controlled-release behavior is crucial for agricultural applications, improving fertilizer efficiency while minimizing environmental pollution caused by excessive urea leaching. Therefore, nanohydroxyapatite-activated carbon composites present a promising approach for developing more efficient and environmentally sustainable slow-release fertilizers.

## Materials and methods

### Materials

Chicken eggshell as a precursor of calcium oxide (CaO). Diamonium hydrogen phosphate (NH_4_)_2_HPO_4_). hydrochloric acid (HCl, 37%), ethanol (C_2_H_5_OH, 100%), p-dimethylaminobenzaldehyde (C_9_H_11_NO), and urea (CO(NH_2_)_2_) were purchased from Merck & Co. Inc, USA. This study used commercial activated carbon (Haycarb AKO 8 × 30, Haycarb PLC, Sri Lanka) derived from steam-activated coconut shell carbon. Distilled water was obtained from the Department of Chemistry, Faculty of Mathematics and Natural Sciences, Universitas Padjadjaran. All chemicals used were of analytical grade and used without further purification.

### Synthesis of nanohydroxyapatite-activated carbon composite

**Fig. 6:**
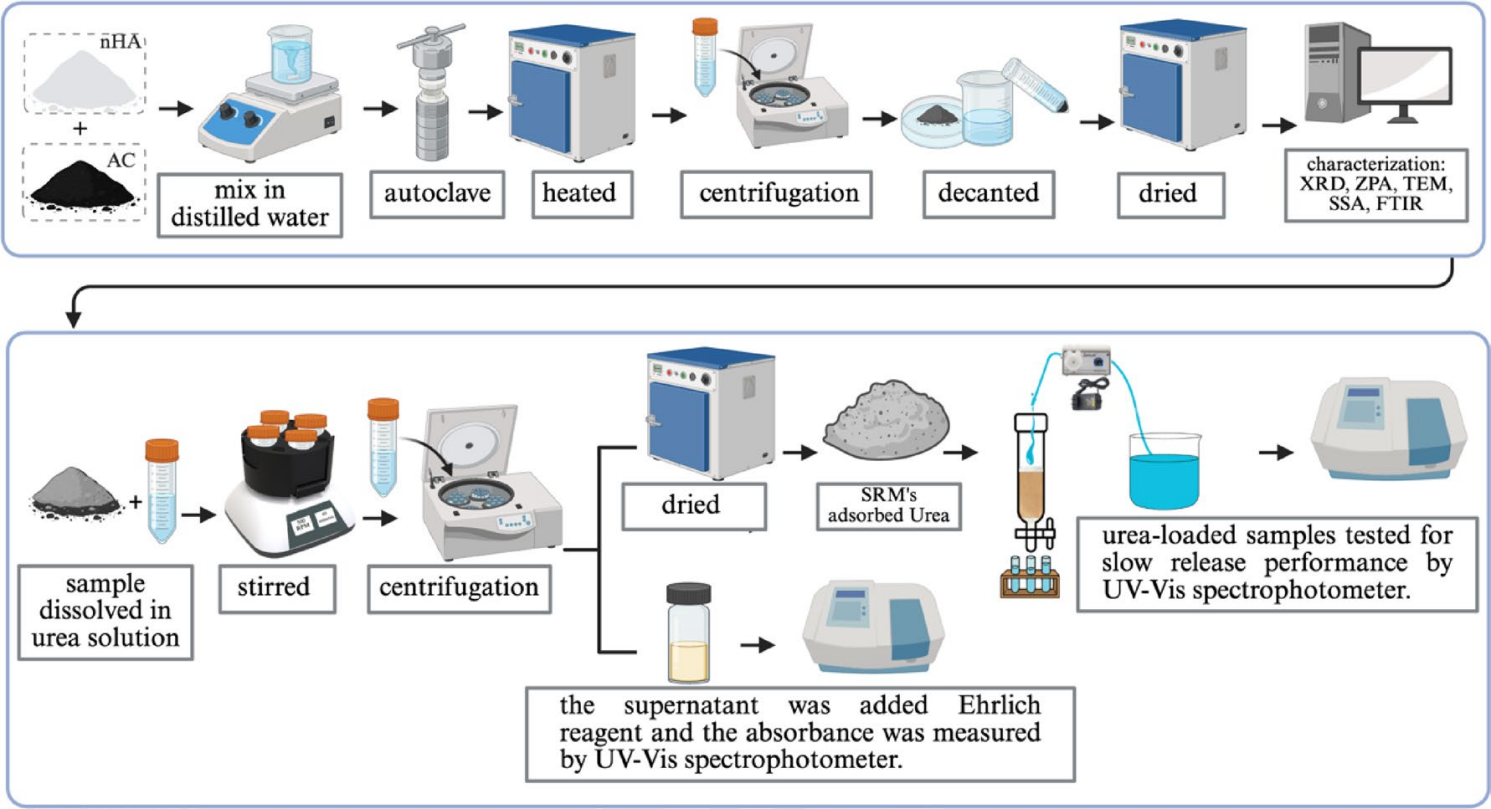
A general scheme summarizing the experimental workflow in this study, including synthesis, characterization, urea adsorption test and urea release evaluation.

This study synthesized composites using a two-stage hydrothermal process in a Teflon-lined autoclave maintained at 230°C for 48 h. In the first stage, nanohydroxyapatite (nHA) was produced from calcium oxide (CaO) and diammonium hydrogen phosphate, with a Ca/P molar ratio of 1.67. The mixture was combined with 100 mL of distilled water, and the pH was adjusted to 9. Next, the mixture was placed into the autoclave and heated in an oven at 230°C for 48 h. The resulting HA solution was filtered and washed with distilled water using a centrifuge until it reached a pH of 7. Finally, the nHA was dried in an oven at 110°C for 2 h^41^. In the second hydrothermal process, nHA was mixed with AC in a specific ratio into 100 mL of distilled water. The wt% ratios of HA to AC were 75:25, 50:50, and 25:75, respectively, referred to as HC1, HC2, and HC3. The mixture was then put into an autoclave and heated at 230°C for 48 h. The synthesized product was filtered and washed with distilled water, then dried at 110°C for 2 h. The entire workflow, including synthesis, characterization, adsorption urea test and urea release evaluation, is briefly illustrated in Fig. 6.

### Characterizations

The synthesized nHA, AC, and composites were characterized using various analytical techniques to determine their structural, morphological, and surface properties. X-ray diffraction (XRD) analysis determined each sample’s structure and crystal size. XRD measurements were performed using a Bruker D8 Advance diffractometer with Cu Kα radiation (λ = 1.54 Å) over a diffraction angle range (2θ) of 10°–70° at a scan speed of 2°/min. Zeta potential analysis was carried out using a Horiba SZ-100 Zeta Potential Analyzer to assess the surface charge of each sample. The morphology and surface characteristics of the nanocomposites were examined using high-resolution transmission electron microscopy (HRTEM; Thermo Fisher Scientific FEI Tecnai G2 Supertwin). Surface area analysis was conducted using a Quantachrome Quadrasorb-Evo surface area analyzer, employing the Brunauer-Emmett-Teller (BET) method. Fourier transform infrared (FTIR) spectroscopy was performed using a PerkinElmer Spectrum 100 spectrometer to analyze nHA, AC, and composites before and after urea adsorption. The analysis was conducted using the potassium bromide (KBr) pellet technique.

### Evaluation of SRMs’ adsorption capacity for Urea

To determine the adsorption capacity of urea, 0.5 g of nHA, AC, and composites was added to 25 mL of a 0.5 M urea solution. The mixture was stirred using an incubator shaker at 500 rpm for 1 h. It was then centrifuged at 8000 rpm for 10 min, after which the supernatant was separated, and the precipitate was dried at 80°C for 24 h^6^.The adsorption of urea in the supernatant was measured using a UV-Vis spectrophotometer (Genesys 10 S UV-Vis, Thermo Scientific, USA) at a wavelength of 417 nm with the Ehrlich reagent. The reagent consisted of 2 mL of 4% *p*-dimethylaminobenzaldehyde and 0.1 mL of 4% HCl in 10 mL of 96% ethanol. To determine the urea concentration, 0.5 mL of the reagent was added to 2 mL of the sample, producing a yellow solution. The amount of urea adsorbed was calculated based on the difference in urea concentration before and after adsorption^41,42^. The adsorption capacity of urea’s slow-release materials (SRMs) was calculated by comparing the solution concentration before and after adsorption. All adsorption tests were duplicated, and results were presented as mean ± standard deviation. The adsorption capacity (q_t_) was determined using the following equation:

1 $$\:{q}_{t}=\frac{\left({C}_{0}-{C}_{\text{t}}\right)V}{m}$$.

Where *q*_t_ is the adsorption capacity (g_urea_/g_SRM_), *C*_0_ is the initial concentration (mol/L), *C*_t_ is the concentration after adsorption (mol/L), *V* is the volume of the solution (L), and *m* is the mass of the adsorbent (g).

### Evaluation of slow-release properties for Urea

**Fig. 7:**
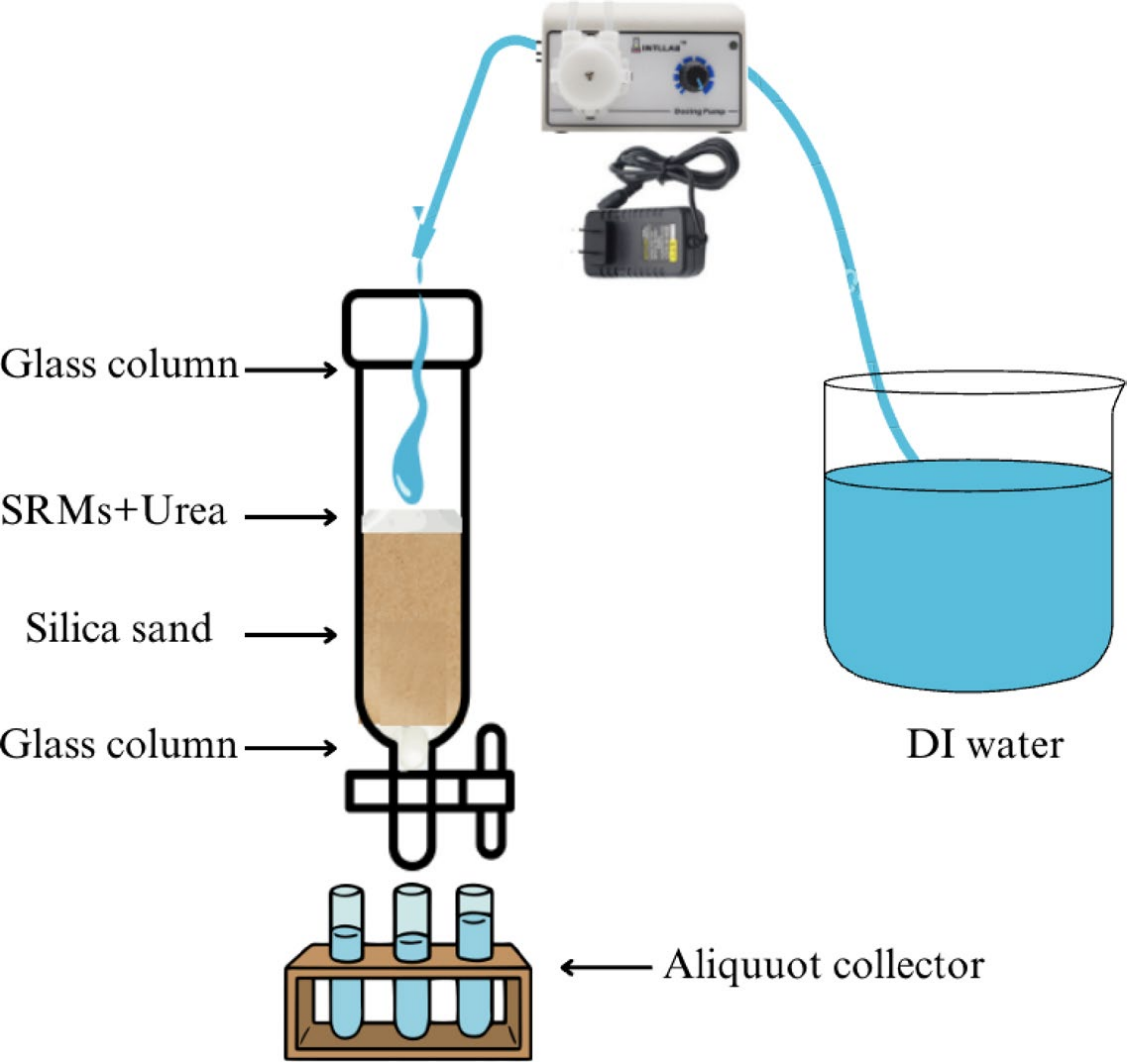
The arrangement illustration of slow-release performance test for SRMs-Urea.

Silica sand (20–40 mesh) was added into a glass column (i.d. 15 × 300 mm) with a silica sand height of 100 mm. Subsequently, deionized water was flowed into the glass column until it reached the top layer of silica sand. Further, urea (U) was introduced onto the top layer of the silica sand. After that, deionized water was pumped into the column with a flow rate of 5 mL/min using a peristaltic pump. The eluted solution was collected with an interval of 15-minute intervals for 240 min. The concentration of urea in the collected samples was determined using the Ehrlich reagent method, with absorbance measured at a wavelength of 417 nm using a UV–Vis spectrophotometer (Genesys 10 S UV–Vis, Thermo Scientific Corp., USA). For comparison, urea-loaded samples (U-nHA, U-AC, U-HC1, U-HC2, and U-HC3) were treated using a similar procedure. All release tests were duplicated, and results were presented as mean ± standard deviation. The illustration of the slow-release urea performance test for the SRM samples is presented in Fig. 7.

## Conclusions

Nanohydroxyapatite-activated carbon composites have been successfully synthesized using a two-stage hydrothermal method, as confirmed by XRD, FTIR, and TEM characterization. XRD diffraction patterns revealed the presence of the hydroxyapatite phase, with no significant changes in its crystalline structure after the incorporation of activated carbon. FTIR spectra further confirmed the interaction between nanohydroxyapatite and activated carbon, indicated by shifts in the absorption bands of their typical functional groups. TEM analysis showed that the nanohydroxyapatite particles were uniformly distributed within the activated carbon matrix, resulting in nanocomposites with a homogeneous morphology. Urea adsorption and release studies demonstrated that nanocomposites with a higher nanohydroxyapatite content exhibited enhanced adsorption capacity, with the HC1 composite (75% nHA: 25% AC) showing the slowest urea release compared to other samples. This suggests that combining nanohydroxyapatite and activated carbon improves the material’s effectiveness as a carrier for urea in slow-release fertilizers. Activated carbon helps control the gradual release of urea, making it an essential component in the nanocomposite’s functionality. Therefore, the nanohydroxyapatite-activated carbon nanocomposite synthesized in this study shows significant potential as a slow-release fertilizer material, offering an efficient and sustainable approach to nutrient delivery.

## Electronic supplementary material

Below is the link to the electronic supplementary material.

Supplementary Material 1

## Data Availability

The datasets used and/or analyzed during the current study are available from the corresponding author on reasonable request.
