# Supplementary material for: Hydrothermal synthesis of nanohydroxyapatite-activated carbon composites and its slow-release performance for urea
Source: Sci Rep. 2025 Jul 19;15:26208. doi: 10.1038/s41598-025-09023-w (PMC12276313; doi:10.1038/s41598-025-09023-w)
Supplement: Supplementary file 1 — Supplementary Material 1 [file 41598_2025_9023_MOESM1_ESM.docx]

**Supplementary Material**

**Hydrothermal Synthesis of Nanohydroxyapatite-Activated Carbon Composites and Its Slow-Release Performance for Urea**

Sabila Aulia Hemzah ^a^, Irwan Kurnia ^a, f, *,^ Diana Rakhmawaty Eddy ^a, f^, Bedah Rupaedah ^b, f^, Suryana ^c^, Azman Bin Ma'Amor ^d^, Guoqing Guan ^e^, Atiek Rostika Noviyanti ^a, f, *^

^a^ Department of Chemistry, Faculty of Mathematics and Natural Sciences, Universitas Padjadjaran, Sumedang 45363, Indonesia

^b^ Research Center for Applied Microbiology, National Research and Innovation Agency, Bogor 16911, Indonesia

^c^ Department of Biology, Faculty of Mathematics and Natural Sciences, Universitas Padjadjaran, Sumedang 45363, Indonesia

^d^ Department of Chemistry, Universiti Malaya, Kuala Lumpur 50603, Malaysia

^e^ Institute of Regional Innovation, Hirosaki University, Hirosaki 036-8561, Japan

^f^ Research Collaboration Center for Microbial Nanomaterials between BRIN and Universitas Padjadjaran, Sumedang 45363, Indonesia

*Corresponding author: E-mail address: atiek.noviyanti@unpad.ac.id (A. R. Noviyanti); [irwan.kurnia@unpad.ac.id](mailto:irwan.kurnia@unpad.ac.id) (I. Kurnia); Tel: +62-22-7794391


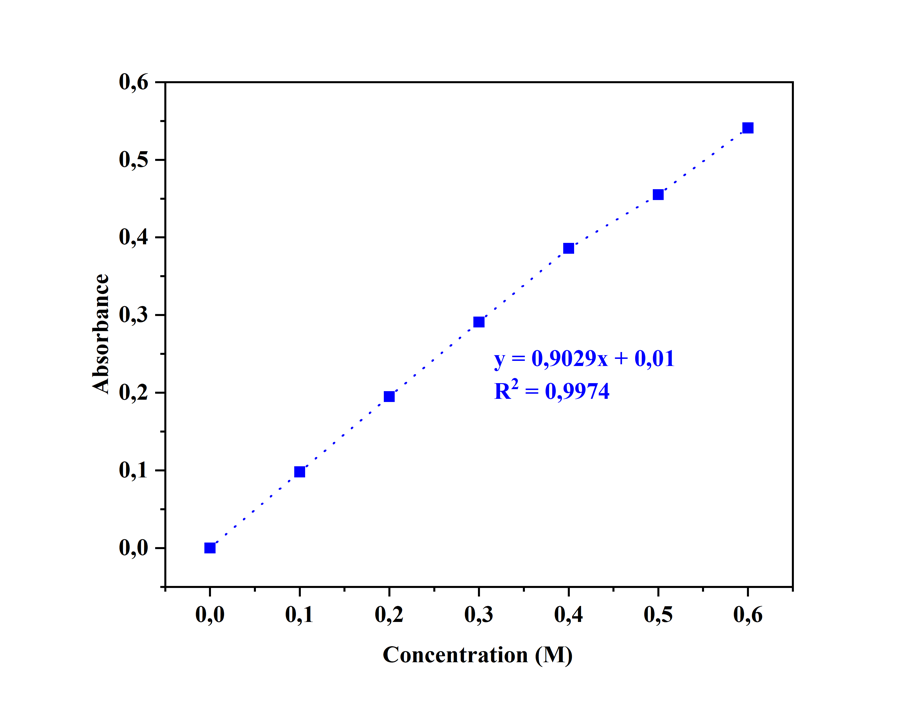


**Figure S1.** External standard curve of urea solution (0; 0.1; 0.2; 0.3; 0.4; 0.5; and 0.6 M).


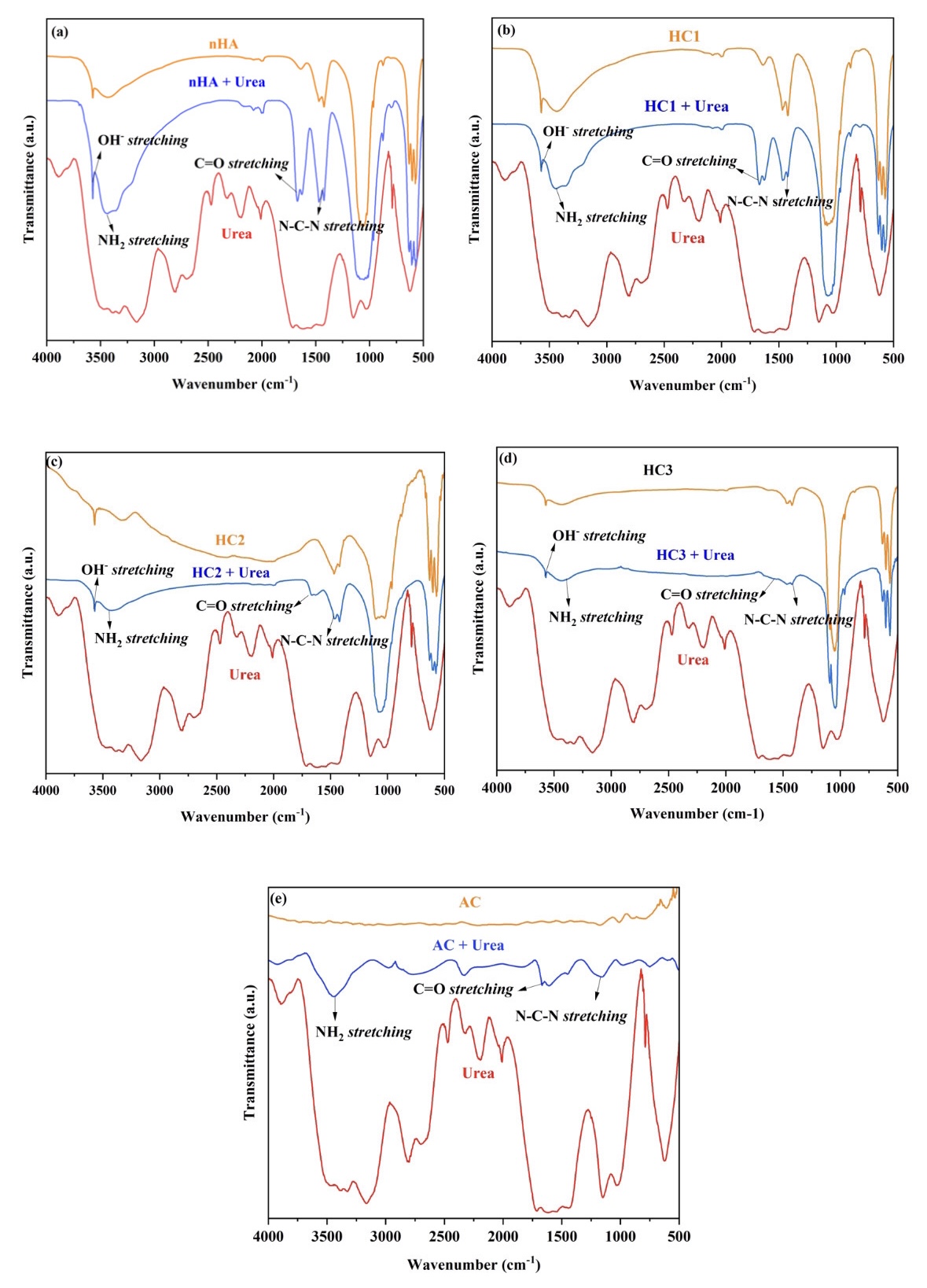


**Figure S2.** FTIR spectra before and after urea adsorption of (a) nHA, (b) HC1, (c) HC2, (d) HC3, and (e) AC.

**
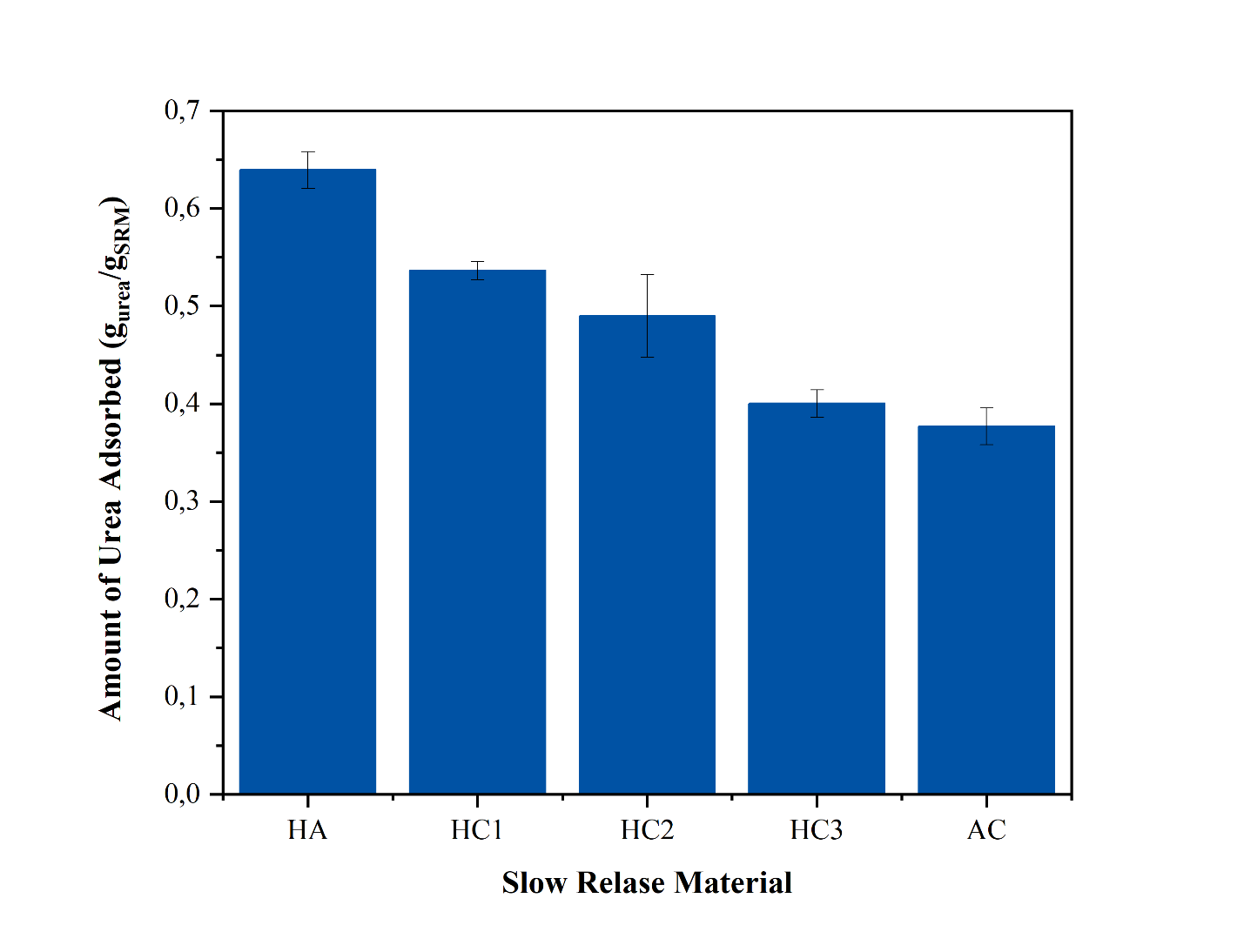
**

**Figure S3.** Adsorption performance of urea onto SRM (U, U-HA, U-AC, U-HC1, U-HC2, and U-HC3), presented as mean ± standard deviation from duplicate experiments.

| Composition | Wave number (cm^-1^) | | | | | | | |
| --- | --- | --- | --- | --- | --- | --- | --- | --- |
|  | *Free* O-H *streching* | N-H *streaching* | C$=$O streaching | N-C-N *stretching* | CO_3_^2-^*streaching* | O-H bending | P-O *streaching* | P-O *bending* |
| Urea | - | 3475 | 1716 | 1430 | - | - | - | - |
| nHA | 3571 | - | - | - | 1422 | 631 | 1056 | 603 |
| nHA-Urea | 3571 | 3451 | 1671 | 1469 | - | 630 | 1048 | 608 |
| HC1 | 3571 | - | - | - | 1421 | 630 | 1079 | 602 |
| HC1-Urea | 3571 | 3454 | 1671 | 1466 | - | 632 | 1075 | 599 |
| HC2 | 3571 | - | - | - | 1421 | 630 | 1052 | 602 |
| HC2-Urea | 3572 | 3436 | 1665 | 1463 | - | 630 | 1080 | 597 |
| HC3 | 3572 | - | - | - | 1422 | 633 | 1089 | 602 |
| HC3-Urea | 3572 | 3442 | 1662 | 1463 | - | 630 | 1091 | 603 |
| AC | - | - | 1668 | - | - | 660 | - | - |
| AC-Urea | - | 3441 | 1663 | 1468 | - | 642 | - | - |

**Table S1.** Comparison of FTIR spectra of slow-release materials (SRMs) before and after urea

**Table S2.** Parameter of adsorption capacity of slow-release materials (SRMs) for urea.

| Sample | *C*_0_ (mol/L) | C_t_ | Adsorbed urea (%) | *q_t_*  (g_urea_/g_SRM_) |
| --- | --- | --- | --- | --- |
| nHA | 0.5 | 0.30 | 39.08 | 0.5863 |
| HC1 | 0.5 | 0.32 | 36.20 | 0.5431 |
| HC2 | 0.5 | 0.35 | 30.66 | 0.4600 |
| HC3 | 0.5 | 0.36 | 27.34 | 0.4102 |
| KA | 0.5 | 0.38 | 24.24 | 0.3637 |

C_0_ : initial concentration of urea. C_t_ : concentration of urea adsorption; nHA = nanohydroxyapatite, HK = composite of HA and AC, AC = activated carbon.

**Table S3.** Release rate equations, urea release rates, and estimated release times of various urea loaded-slow release materials (SRMs) .

| Sample | Release Rate Equation | Release Rate of Urea | Estimated Release Time |
| --- | --- | --- | --- |
| nHA | y = 0.2454x + 0.5508 | 1521.4 µg_urea_min^-1^ | 405 minutes  (6.7 hours) |
| HC1 | y = 0.0611x + 1.119 | 333.8 µg_urea_min^-1^ | 1.618 minutes  (27 hours) |
| HC2 | y = 0.1716x + 0.7356 | 847.1 µg_urea_min^-1^ | 578 minutes  (10 hours) |
| HC3 | y = 0.2088x + 0.8307 | 848.8 µg_urea_min^-1^ | 475 minutes  (8 hours) |
| AC | y = 0.3962x + 3.3841 | 1512.2 µg_urea_min^-1^ | 244 minutes  (4 hours) |

**Table S4.** Mean ± standar deviation of urea adsorption capacity from each sample.

| No | Sample | Mean ± Standar Deviation |
| --- | --- | --- |
| 1 | HA | 0.639 g_urea_g^-1^_SRM_ ± 0.02 |
| 2 | HC1 | 0.536 g_urea_g^-1^_SRM_ ± 0.01 |
| 3 | HC2 | 0.490 g_urea_g^-1^_SRM_ ± 0.04 |
| 4 | HC3 | 0.400 g_urea_g^-1^_SRM_ ± 0.01 |
| 5 | AC | 0.377 g_urea_g^-1^_SRM_ ± 0.02 |

**Table S5.** Mean ± standar deviation of urea release percentage from each sample.

| **Time** | **HA** | **HC1** | **HC2** | **HC3** | **AC** |
| --- | --- | --- | --- | --- | --- |
| 0 | 0.0 ± 0.0 | 0.0 ± 0.0 | 0.0 ± 0.0 | 0.0 ± 0.0 | 0.0 ± 0.0 |
| 15 | 3.6 ± 0.0 | 1.4 ± 0.6 | 3.4 ± 0.6 | 4.0 ± 0.0 | 8.3 ± 0.1 |
| 30 | 7.7 ± 0.1 | 2.6 ± 1.2 | 6.0 ± 0.8 | 7.3 ± 0.2 | 15.1 ± 0.5 |
| 45 | 11.5 ± 0.2 | 3.9 ± 1.7 | 8.7 ± 1.0 | 10.6 ± 0.3 | 21.9 ± 0.5 |
| 60 | 15.6 ±0.2 | 5.1 ± 1.9 | 11.2 ± 1.1 | 13.6 ± 0.5 | 28.1 ± 0.7 |
| 75 | 19.1 ± 0.3 | 6.2 ± 2.2 | 13.6 ± 1.2 | 16.7 ± 0.7 | 34.1 ± 0.7 |
| 90 | 22.9 ± 0.6 | 7.2 ± 2.2 | 16.2 ± 1.5 | 19.7 ± 0.9 | 39.9 ± 0.7 |
| 105 | 26.7 ± 1.0 | 8.2 ± 2.2 | 18.8 ± 1.8 | 22.8 ± 1.0 | 45.9 ± 0.7 |
| 120 | 30.3 ± 1.2 | 9.0 ± 2.1 | 21.4 ± 2.2 | 25.9 ± 1.2 | 51.9 ± 0.6 |
| 135 | 33.9 ± 1.5 | 9.9 ± 2.1 | 24.0 ± 2.4 | 29.0 ± 1.3 | 57.9 ± 0.2 |
| 150 | 37.7 ± 1.9 | 10.7 ± 2.1 | 26.7 ± 2.6 | 32.1 ± 1.7 | 63.9 ± 0.4 |
| 165 | 41.3 ± 2.2 | 11.5 ± 2.2 | 29.2 ± 2.6 | 35.1 ± 1.8 | 69.7 ± 1.2 |
| 180 | 45.0 ± 2.5 | 12.3 ± 2.3 | 31.7 ± 2.6 | 38.3 ± 2.1 | 75.3 ± 2.0 |
| 195 | 48.6 ± 2.9 | 13.0 ± 2.4 | 34.2 ± 2.7 | 41.5 ± 2.3 | 80.6 ± 2.7 |
| 210 | 52.1 ± 3.5 | 13.6 ± 2.5 | 36.7 ± 2.9 | 44.7 ± 2.6 | 85.9 ± 3.2 |
| 225 | 55.4 ± 3.9 | 14.3 ± 2.6 | 39.2 ± 3.0 | 47.9 ± 3.0 | 91.0 ± 3.8 |
| 240 | 58.3 ± 4.3 | 14.9 ± 2.6 | 41.6 ± 3.3 | 50.8 ± 3.4 | 96.4 ± 4.4 |
